# Preventive Aerobic Training Protects Against Doxorubicin-Induced Cardiotoxicity by Preserving Redox Status and Attenuating Cardiac Stress-Related Signaling

**DOI:** 10.3390/cells15050408

**Published:** 2026-02-26

**Authors:** Paola Victória da Costa Ghignatti, Rafael Aguiar Marschner, Rafael Teixeira Ribeiro, Vitor Gayger-Dias, Vanessa-Fernanda Da Silva, Luciele Varaschini Teixeira, Simone Wajner, Maximiliano Isoppo Schaun, Carlos-Alberto Gonçalves, Patrícia Sesterheim

**Affiliations:** 1 Institute of Cardiology of Rio Grande do Sul, University Foundation of Cardiology (IC/FUC), Porto Alegre 90040-371, RS, Brazil; vicghignatti@gmail.com (P.V.d.C.G.); luciele.sm@gmail.com (L.V.T.); dr.maxschaun@gmail.com (M.I.S.); 2 Biochemistry Department, Basic Sciences Institute of Health, Universidade Federal do Rio Grande do Sul (UFRGS), Porto Alegre 91501-970, RS, Brazil; rafaelrated@hotmail.com (R.T.R.); vitor.dias@ufrgs.br (V.G.-D.); vanessafernanda171191@gmail.com (V.-F.D.S.); casg@ufrgs.br (C.-A.G.); 3 Endocrine Division, Hospital de Clínicas de Porto Alegre, Universidade Federal do Rio Grande do Sul (UFRGS), Porto Alegre 90035-003, RS, Brazil; simone.wajner@ufrgs.br

**Keywords:** aerobic training, cardiotoxicity, doxorubicin, oxidative stress, heart

## Abstract

Doxorubicin (DOX) is a highly effective chemotherapeutic agent whose clinical use is limited by dose-dependent cardiotoxicity associated with oxidative stress, inflammation, and cellular stress responses. Here, we investigated whether preventive aerobic training could protect against DOX-induced cardiac injury in Wistar rats. Animals were assigned to sedentary control (C), sedentary DOX (D), trained control (CT), and trained DOX (DT) groups. The moderate-intensity (~50–80% maximal exercise test) treadmill protocol (40 min/day, 4 days/week for 4 weeks) was performed before intraperitoneal administration of DOX (4 mg/kg, weekly for 4 weeks) or saline. Preventive training markedly improved exercise capacity (*p* < 0.001) and attenuated oxidative damage, maintaining antioxidant enzyme activity (GR, SOD) at control levels (*p* > 0.05). DOX significantly upregulated cardiac *IL-6* and *IL-1β* expression (*p* < 0.01), while trained animals preserved *IL-1β* expression similar to controls (*p* > 0.99). In parallel, DOX increased cardiac *HIF-1* expression (*p* < 0.05), indicating activation of hypoxia- and stress-related signaling pathways, an effect that was attenuated by preventive training (*p* > 0.99). DOX-induced cardiac atrophy was evidenced by reduced left ventricular mass (*p* < 0.001), which was partially prevented by training (*p* < 0.05). Although hematological toxicity persisted, preventive aerobic exercise effectively counteracted DOX cardiotoxicity by restoring redox homeostasis, dampening inflammation, and limiting apoptotic signaling. Collectively, these findings highlight exercise preconditioning as a promising non-pharmacological strategy in cardio-oncology to mitigate chemotherapy-associated cardiac injury.

## 1. Introduction

Cancer and cardiovascular diseases (CVDs) remain the two leading causes of death worldwide [1]. Advances in cancer therapy have significantly improved survival rates; however, many treatments, particularly anthracyclines, exert detrimental effects on the cardiovascular system [2]. Consequently, CVD has emerged as a major cause of morbidity and mortality among cancer survivors [3], underscoring the urgent need for strategies that protect the heart without reducing the efficacy of anticancer therapy. Among the chemotherapeutic agents, anthracyclines, particularly doxorubicin (DOX), are highly effective [4,5], but they are limited by their cardiotoxic effects, which can manifest as reduced left ventricular ejection fraction (LVEF) and heart failure, complicating their clinical use [6].

The search for cardioprotective strategies that can mitigate the harmful effects of DOX without compromising its antitumor efficacy is crucial. While pharmacological interventions like dexrazoxane and beta-blockers have been explored, non-pharmacological approaches particularly physical exercise have gained prominence due to their proven cardiovascular benefits [7,8,9]. Regular aerobic training promotes myocardial strengthening, improves cardiac efficiency, reduces blood pressure, and stimulates angiogenesis [10,11], contributing to a cardioprotective phenotype and supporting cardiovascular health during and after chemotherapy.

At the molecular level, exercise stimulates endogenous antioxidant defenses through Nrf2 activation and controlled production of reactive oxygen species (ROS), leading to improved redox homeostasis and attenuation of inflammation [12,13]. These beneficial effects make physical exercise a promising strategy for mitigating tissue damage associated with various clinical conditions, including chemotherapy-induced cardiotoxicity.

Although accumulating evidence suggests that aerobic exercise may attenuate DOX-induced cardiotoxicity, most previous studies have primarily focused on specific molecular pathways or isolated functional assessments. Given the methodological heterogeneity across experimental models, including differences in exercise protocols, DOX regimens, timing of interventions, and outcome measures, further integrative in vivo investigations are necessary to consolidate and strengthen the translational relevance of this strategy.

Accordingly, the present study was designed to investigate the effects of preventive aerobic training on cardiac structure, functional parameters, and redox-related signaling in a physiologically intact in vivo model of chronic DOX-induced cardiotoxicity. Rather than targeting a single regulated cell death or inflammatory pathway, we aimed to evaluate the global cardiac phenotype following exercise preconditioning. We hypothesized that preventive aerobic training would attenuate DOX-induced cardiac alterations by promoting redox balance and preserving left ventricular mass (LVM).

## 2. Materials and Methods

### 2.1. Animals and Procedures

Male Wistar Kyoto rats, 8 weeks of age (weighing between 100 and 150 g), were used in the experiments. All procedures and experiments involving animals followed the guidelines of the Brazilian Society for Laboratory Animal Science (SBCAL/COBEA). This study adhered to the Animal Research: Reporting of In Vivo Experiments (ARRIVE) guidelines [14]. The Research Ethics Committee and the Animal Ethics Committee of our institution approved the study (Protocol No. UP5517/18).

**Figure 1:**
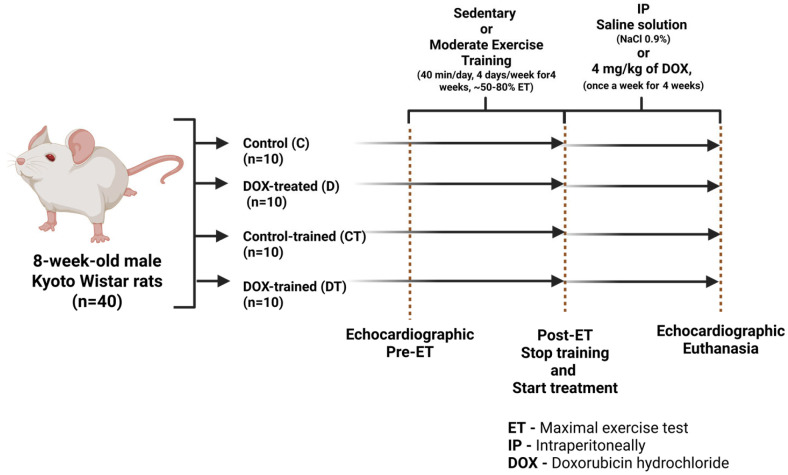
Experimental design. The arrows indicate the timeline. At the end of the protocol, the animals were weighed, and ventricular thickness was assessed by echocardiography 1 week after the administration of the final cumulative dose of doxorubicin (DOX). After an additional 24 h, the animals were lightly anesthetized with isoflurane and sacrificed by decapitation.

All animals received a standard diet and water *ad libitum* throughout the experiment. Environmental conditions were rigorously controlled, ensuring a 12 h light/dark cycle, a temperature of 22 ± 1 °C, and an air exhaust system. Sample size was calculated as described by Ghignatti et al. (2022) [15], since the studies are interconnected, with ten animals used per group. Forty Wistar Kyoto rats were randomly allocated to four experimental groups (*n* = 10 per group): sedentary control (C), sedentary doxorubicin (D), trained control (CT), and trained doxorubicin (DT). Randomization was performed by an external technician from the animal facility using a simple random allocation procedure, without stratification based on baseline parameters. At the beginning of the experimental protocol, animals presented similar age, body weight, and left ventricular mass ranges, as detailed in Supplementary Table S1. Groups C and D remained sedentary for four weeks, after which group C received weekly intraperitoneal injections of 0.9% NaCl for four weeks, while the group D was treated with weekly intraperitoneal injections of doxorubicin (DOX, doxorubicin hydrochloride, Fauldoxo^®^, Libbs, São Paulo, Brazil) at a dose of 4 mg/kg, totaling a cumulative dose of 16 mg/kg [15,16]. The CT and DT groups underwent an aerobic training protocol for four weeks, with four sessions per week, prior to receiving either intraperitoneal injections of 0.9% NaCl (CT group) or DOX (DT group), as described above. The experimental design is depicted in Figure 1.

### 2.2. Maximal Exercise Test and Physical Training

Maximal exercise tests (ETs) were conducted before and after the training period [17]. Following a three-day treadmill adaptation (0.3 km/h for 15 min/day; week 0), animals performed an incremental ET starting at 0.3 km/h, with 0.3 km/h increases every 3 min until voluntary exhaustion. The aerobic training protocol was based on ~50–80% (moderate intensity) of the maximal speed reached during ET, progressing in duration and intensity over 4 weeks (40 min/day, 4 days/week; weeks 1 to 4) [17]. Sedentary animals also underwent adaptation and ET procedures but remained in cages throughout the training period. To control for environmental stress, their cages were kept in the training room.

### 2.3. Doxorubicin-Induced Cardiomyopathy

After the first 4 weeks of the protocol (sedentary or physical training), the animals received weekly intraperitoneal injections of either saline solution (NaCl 0.9%) or 4 mg/kg of doxorubicin (DOX, doxorubicin hydrochloride, Fauldoxo^®^, Libbs, São Paulo, Brazil) for four weeks (weeks 5 to 8), totaling a cumulative dose of 16 mg/kg [15]. The animals were monitored for signs of pain and toxicity, such as lethargy, irregular breathing, weight loss, changes in fur, and ocular discharge. Moreover, the DOX-induced cardiomyopathy protocol used in this study has previously been shown by our group [16] to reduce LVEF—the gold standard parameter for diagnosing and monitoring cardiotoxicity.

### 2.4. Evaluation of Ventricular Thickness

Echocardiography (two-dimensional and M-mode) was performed one week after the fourth injection of saline or DOX (week 9). The animals were anesthetized with a mixture of 2–3% isoflurane (100%, 1 mL/mL, Isoforine, Cristália, São Paulo, Brazil) in 100% oxygen and positioned in the left lateral decubitus. The echocardiographic analyses were conducted by a blinded examiner using the EnVisor echocardiograph (Philips, Andover, MA, USA) with a 12 MHz transducer. Measurements of the anterior left ventricular wall thickness (AWT), posterior wall thickness (PWT), and final diastolic diameter of the left ventricle (LVEDD) were obtained. These measurements were used to assess LVM, which was calculated using the following formula, assuming a spherical left ventricular geometry, validated in rats: LVM (g) = 1.04 × [(LVEDD + AWT + PWT)^3^ − LVEDD^3^] [18,19].

### 2.5. Euthanasia and Tissue Collection

One week after the last intraperitoneal injection of DOX/saline and after final echocardiography (week 9), the animals were anesthetized with a mixture of 2–3% isoflurane (100%, 1 mL/mL, Isoforine, Cristália) in 100% oxygen until they ceased to resist, prior to euthanasia. After blood collection, the animals were euthanized by decapitation for heart collection. After removal, the hearts were washed with saline solution and weighed individually.

### 2.6. Blood Analysis

Total blood was collected, in tubes with 10% EDTA, by cardiac puncture. Erythrocytes were manually counted using a hemocytometer by macrodilution in 0.9% saline solution. Hematocrit was determined using the microhematocrit technique, followed by reading from specific tables; hemoglobin was obtained by calculation. All analyses were performed as previously described [20,21].

### 2.7. Real-Time PCR

Total RNA was extracted from tissues using the Trizol method, and cDNA was synthesized (SuperScript First-Strand Synthesis System for RT-PCR; Invitrogen, Carlsbad, CA, USA), followed by real-time PCR with SYBR Green PCR Power Up (Applied Biosystems, Waltham, MA, USA) on the ABI Prism Vii7 Sequence Detection System (Applied Biosystems). The r^2^ value was greater than 0.99, and amplification efficiency ranged from 80% to 100%. Samples were measured by relative quantification (change in expression in the experimental group versus sham; untreated group versus treated group). The primers used are described in Table 1.

### 2.8. Carbonyl Measurement

Carbonyl content was determined as described by Zanatta et al. (2013) [22], based on the reaction with 2,4-dinitrophenylhydrazine and absorbance measured at 370 nm. The results were calculated using the molar extinction coefficient (ε 370 nm = 21,000,000 M^−1^·cm^−1^) and expressed as nmol carbonyl/mg protein.

### 2.9. Malondialdehyde Levels

Malondialdehyde (MDA) levels were measured via TBARS assay [23]. Fluorescence was detected at 515 nm (excitation) and 553 nm (emission). A calibration curve with 1,1,3,3-tetramethoxypropane was used, and results were expressed as nmol MDA/mg protein.

### 2.10. Sulfhydryl Content

Thiol content was quantified using the DTNB method (Aksenov and Markesbery), with absorbance read at 412 nm [24]. The results were expressed as nmol TNB/mg protein.

### 2.11. Antioxidant Defenses

The levels of reduced glutathione (GSH) levels were determined fluorometrically using o-Phthalaldehyde, with excitation at 350 nm and emission at 420 nm [25]. Results were expressed in nmol/mg protein.

### 2.12. Glutathione Reductase (GR) and Superoxide Dismutase (SOD) Activities

GR activity was evaluated by monitoring NADPH consumption at 340 nm [26] and expressed as U/mg protein. SOD activity was assessed according to Marklund [27], with absorbance measured at 420 nm.

### 2.13. Statistical Analysis

Statistical analyses were performed using GraphPad Prism software version 8.0.1 (San Diego, CA, USA). Normality of data was assessed using the Shapiro–Wilk test. All variables followed a parametric distribution. The results are presented as mean ± standard deviation (SD). Comparisons between experimental groups were assessed by one-way or two-way ANOVA, followed by Tukey or Bonferroni post hoc tests. Statistical significance was defined as *p* < 0.05.

## 3. Results

### 3.1. Effects Induced by Physical Training and Doxorubicin-Induced Cardiomyopathy

Preventive aerobic training did not affect overall body weight; however, animals in the DOX-treated groups (D and DT) showed significant weight loss compared to the other groups (*p* < 0.05) (Table 2). Exercise capacity, assessed by maximal running speed in the ET, was significantly improved in trained groups (CT and DT) compared to sedentary controls (C and D) (*p* < 0.001) (Table 2). DOX administration reduced erythrocyte count, hematocrit, and hemoglobin levels (D vs. C, *p* < 0.05) (Table 2). Preventive training (DT) did not mitigate these hematological alterations when compared to C (*p* > 0.05). Notably, isolated training (CT) increased erythrocyte count relative to DT (*p* < 0.01), suggesting a positive effect of exercise independent of DOX exposure. In summary, preventive training could attenuate, but not normalize, specific hematological parameters under DOX exposure.

### 3.2. Physical Training Induces Changes in Nitric Oxide (NO) Production—An Effect Lost with the Use of DOX

**Figure 2:**
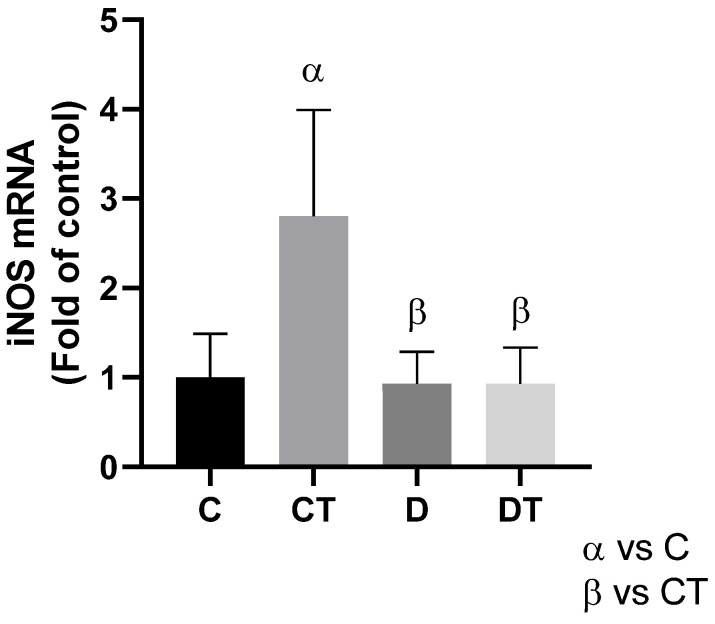
Adaptations of physical training on nitric oxide synthesis through modulation of *iNOS* (inducible nitric oxide synthase) expression. *iNOS* mRNA expression. α = group compared to C (*p* < 0.01); β = group compared to CT (*p* < 0.01); *n* = 10 in each group. C = sedentary control; D = sedentary doxorubicin; CT = trained control; DT = trained doxorubicin.

Physical training resulted in a significant increase in *iNOS* expression in the CT group (*p* < 0.005) compared to the other groups (Figure 2). However, this effect was not observed in the DT group, where *iNOS* levels did not show a significant difference compared to the other groups (*p* > 0.99).

### 3.3. Induction of Pro-Inflammatory Cytokines

**Figure 3:**
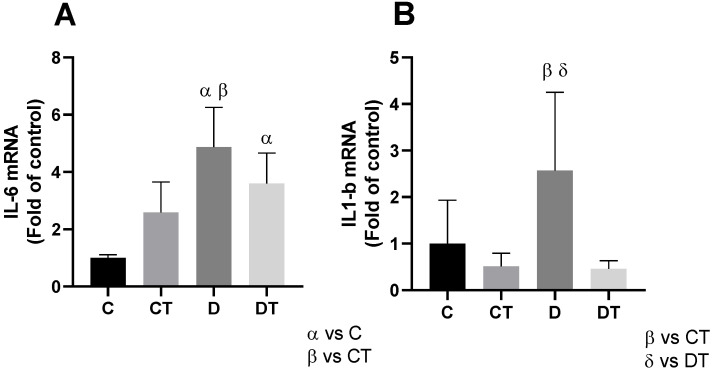
Evaluation of inflammatory markers, *IL-6* (interleukin-6; (**A**)) and *IL-1β* (interleukin-1-beta; (**B**)), in the heart. The expression levels of *IL-6* increased in groups D and DT, while the expression of *IL-1β* was increased only in group D. α = group compared to C (*p* < 0.01); β = group compared to CT (*p* < 0.01); δ = group compared to DT (*p* < 0.01); *n* = 10 in each group. C = sedentary control; D = sedentary doxorubicin; CT = trained control; DT = trained doxorubicin.

DOX administration resulted in a significant increase in pro-inflammatory cytokines. The expression of the *IL-6* gene was markedly higher in the D and DT groups compared to the C group (*p* < 0.01) (Figure 3A). Similarly, the expression of the *IL-1β* gene was significantly increased in the D group compared to the C group (*p* < 0.01). However, prior physical training was able to preserve *IL-1β* gene expression levels in the DT group, which remained similar to those observed in the control group (*p* > 0.99) (Figure 3B).

### 3.4. Redox Status

#### 3.4.1. Protection Against Protein and Lipid Oxidation

**Figure 4:**
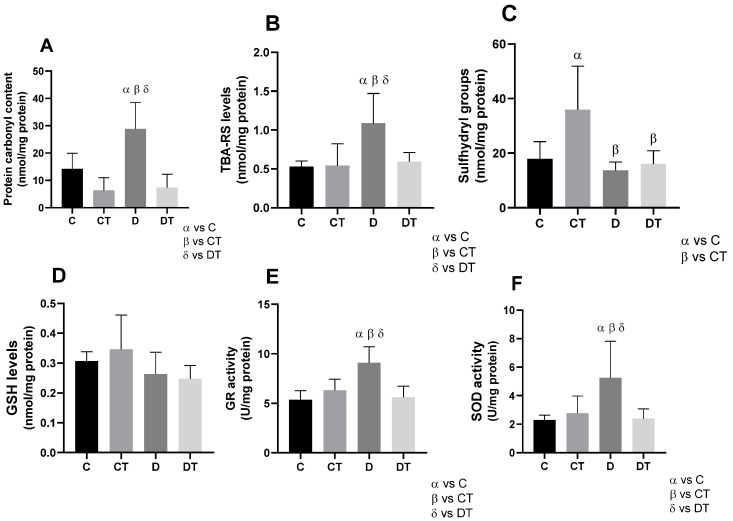
Oxidative stress and antioxidant defense parameters: Carbonyl (**A**); TBARS (thiobarbituric acid reactive substances; (**B**)); sulfhydryl content (**C**); GSH (reduced glutathione; (**D**)); GR (glutathione reductase; (**E**)); and SOD (superoxide dismutase; (**F**)) in the heart. α = group compared to C (*p* < 0.01); β = group compared to CT (*p* < 0.01); δ = group compared to DT (*p* < 0.01); *n* = 10 in each group. C = sedentary control; D = sedentary doxorubicin; CT = trained control; DT = trained doxorubicin.

DOX administration significantly increased protein carbonyls and lipid peroxidation (TBARS) in group D compared to all other groups (*p* < 0.05) (Figure 4A,B). Preventive physical training attenuated these effects, as DT animals exhibited carbonyl and TBARS levels comparable to the control groups (C and CT; *p* = 0.99), indicating the protective role of prior exercise. Sulfhydryl content, indicative of thiol antioxidant status, was significantly elevated in the CT group (*p* < 0.001) (Figure 4C), suggesting enhanced redox buffering with exercise. However, this increase was not preserved in the DT group, implying that DOX blunted the exercise-induced elevation in thiol availability.

#### 3.4.2. Improvement of Antioxidant Profile

No significant differences were observed in GSH levels among the experimental groups (Figure 4D). However, DOX administration led to a marked increase in glutathione reductase (GR) and superoxide dismutase (SOD) activities in group D (*p* < 0.005 and *p* < 0.05, respectively), suggesting a compensatory response to oxidative stress (Figure 4E,F). In contrast, the DT group maintained GR and SOD activities at levels similar to the C and CT groups, indicating that preventive exercise preserved baseline enzymatic antioxidant defenses and may have mitigated redox imbalance induced by DOX.

#### 3.4.3. Hypoxia Levels in Cardiac Tissue and Changes in Left Ventricular Mass

**Figure 5:**
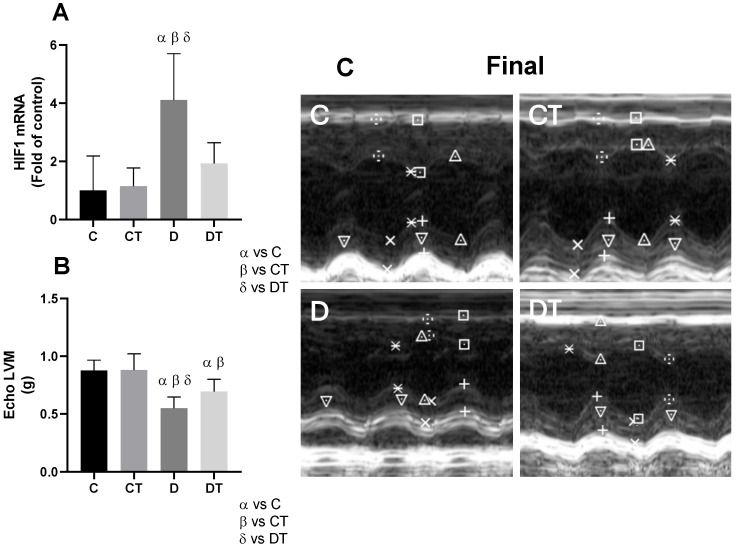
Cardiac stress-related signaling and structural remodeling assessed by final M-mode echocardiography. *HIF-1* (hypoxia-inducible factor 1) mRNA expression (**A**); echocardiographic assessment of left ventricular mass (Echo LVM; (**B**)); and representative cardiac echocardiography images (**C**). (**C**) shows representative M-mode echocardiographic images obtained at the basal level of the left ventricle in the parasternal short-axis view. Symbols in the M-mode images indicate the measurement points used for echocardiographic analysis. The vertical scale represents distance (cm), and the horizontal scale represents time (ms). Measurements were performed according to standardized echocardiographic guidelines. The full, unedited echocardiography images are provided in the Supplementary Materials. α = group compared to C (*p* < 0.01); β = group compared to CT (*p* < 0.01); δ = group compared to DT (*p* < 0.01); *n* = 10 in each group. C = sedentary control; D = sedentary doxorubicin; CT = trained control; DT = trained doxorubicin.

DOX treatment significantly increased *HIF-1* gene expression in the D group (*p* < 0.05), indicating activation of hypoxia- and cellular stress-related signaling pathways (Figure 5A). Preventive physical training mitigated this effect, as *HIF-1* expression in the DT group remained comparable to controls (C and CT; *p* > 0.99).

In parallel, DOX administration resulted in a significant reduction in LVM in both the D and DT groups (*p* < 0.001 vs. controls), but the reduction was significantly less pronounced in the DT group (*p* < 0.05) (Figure 5B), suggesting the protective role of exercise in preserving myocardial structure and cellular viability.

## 4. Discussion

The main findings of this study include (1) the fact that DOX increased pro-inflammatory cytokine levels, protein oxidation, lipid peroxidation, and activation of stress-related molecular pathways. (2) Four weeks of preventive aerobic training improved exercise capacity, enhancing running speed during the exercise test; reduced oxidative damage, attenuating SOD activity and GR levels; and increased sulfhydryl levels. (3) Preventive training contributed to myocardial structural preservation by attenuating stress- and hypoxia-related signaling, as reflected by lower cardiac *HIF-1* gene expression and by the partial preservation of LVM. Taken together, these findings support the hypothesis that preventive physical exercise mitigates DOX-induced cardiac damage primarily through redox homeostasis maintenance and regulation of stress-related signaling pathways, thereby limiting adverse cardiac remodeling and LVM loss.

In a systematic review with meta-analysis [28] developed by our group, the role of physical exercise as a cardioprotective strategy was investigated in experimental rodent models, with a specific focus on its effects on LVEF and left ventricular fractional shortening (LVFS). The results demonstrated that physical exercise in models of DOX-induced cardiotoxicity promoted the preservation of global cardiac function, regardless of protocol duration. These findings are consistent with the data observed in the present study, providing greater support for the hypothesis that physical exercise may serve as an effective prophylactic intervention against the cardiotoxic effects of DOX, enhancing the organism’s particularly the cardiovascular system’s resilience to the insults induced by chemotherapy.

In this context, the modulation of *iNOS* expression observed in our study provides additional insight into the mechanisms underlying DOX-induced oxidative and nitrosative stress. The increased *iNOS* levels after DOX administration reflect exacerbated production of nitric oxide, which, when excessively generated, reacts with superoxide anions to form peroxynitrite, a reactive oxidant known to promote lipid and protein oxidation, mitochondrial dysfunction, and cellular stress signaling [9,29]. Conversely, the attenuation of *iNOS* expression due to training indicates that preventive aerobic training effectively mitigated nitrosative stress, contributing to the maintenance of cellular redox homeostasis. These findings are consistent with previous evidence showing that exercise induces a multifaceted cardioprotective effect, capable of reducing oxidative and nitrosative stress, thereby protecting cardiac cells from DOX-induced injury [10,30,31].

Increasing evidence indicates that the interplay between the accumulation of reactive oxygen species and the activation of inflammatory cytokines critically contributes to the progression of DOX-induced cardiotoxicity, mainly through the sustained imbalance of the redox state and the activation of stress-related molecular pathways [9,32]. This exacerbated inflammatory response can occur acutely or evolve into a chronic inflammatory state, favoring pathological mechanisms such as endothelial dysfunction, platelet activation and disseminated intravascular coagulation [33,34]. These alterations increase the risk of adverse cardiovascular events, including heart failure, and promote myocardial remodeling and fibrosis [35]. Collectively, these processes form a harmful feedback loop in which oxidative and inflammatory damage perpetuate each other, leading to progressive loss of cardiac function.

In this regard, the differential modulation of *IL-1β* and *IL-6* by preventive aerobic training suggests distinct regulatory mechanisms underlying the inflammatory response to DOX. *IL-1β* is strongly associated with processes that are closely linked to mitochondrial dysfunction and redox imbalance, due to inflammasome activation and sustained pro-inflammatory signaling [35], which are known to be attenuated by exercise-induced adaptations [10,30]. Thus, the preservation of *IL-1β* modulation in trained animals may reflect the ability of physical exercise to suppress chronic inflammatory pathways triggered by DOX. In contrast, *IL-6* exhibits a pleiotropic and context-dependent profile, acting not only as a pro-inflammatory cytokine but also as an exercise-induced myokine with metabolic and immunomodulatory functions [10,35]. This dual role may partially explain the absence of a clear exercise-mediated protective effect on *IL-6* levels under DOX exposure, where chemotherapy-induced stress and inflammatory signaling likely predominate [9,36].

Concomitantly, oxidative damage to proteins and lipids is a hallmark of DOX-induced myocardial injury. The scientific literature corroborates that protein carbonylation is an important final byproduct of multiple oxidation pathways that occur in the cell [37], leading to dysfunction of essential proteins and disruption of cellular homeostasis. The accumulation of carbonylated proteins impairs enzymatic activity, compromises structural proteins, and exacerbates cellular stress, thereby contributing to cardiotoxicity [37,38]. In parallel, lipid peroxidation—reflected by increased TBARS levels [39]—compromises cell membrane integrity, alters membrane fluidity, and disrupts intracellular signaling, further aggravating cellular dysfunction [29]. Together, these oxidative processes create a hostile intracellular environment characterized by protein destabilization, membrane damage, and sustained stress signaling, ultimately contributing to myocardial injury and adverse cardiac remodeling [40,41].

In the context of cardiomyocytes, these oxidative alterations are particularly concerning, as DOX has been shown to promote an accelerated aging-like phenotype. Indeed, individuals treated with DOX during childhood often exhibit cellular and functional characteristics resembling premature senescence, including frailty, reduced physiological reserve, increased susceptibility to severe diseases, and diminished muscle strength [42]. In addition, DOX activates stress-responsive signaling pathways involved in cellular adaptation and survival under adverse conditions, including those mediated by *HIF-1*. In the present study, the increased cardiac *HIF-1* expression observed in the D group likely reflects heightened cellular stress and activation of hypoxia- and stress-related signaling pathways, consistent with previous evidence linking *HIF-1* overexpression to mitochondrial dysfunction, metabolic dysregulation, and impaired cellular homeostasis [41,42,43]. Given that classical apoptotic markers (e.g., Bax, Bcl-2, caspase-3) were not assessed, our findings should not be interpreted as direct evidence of apoptotic cell death, but rather as an indication of enhanced cellular stress signaling. Importantly, the attenuation of *HIF-1* expression in the DT group suggests that preventive aerobic training modulates these stress-responsive pathways, thereby contributing to cardiomyocyte structural and functional preservation.

In this scenario, hypoxia-related signaling pathways in the myocardium may be impacted by alterations in redox homeostasis [9]. Sustained activation of *HIF-1* under DOX-induced stress has been associated with metabolic reprogramming, mitochondrial dysfunction, and activation of catabolic pathways that contribute to adverse cardiac remodeling and atrophy [42,43]. Accordingly, the reduced *HIF-1* expression observed in trained animals may reflect improved metabolic efficiency and attenuation of pathological remodeling, contributing to the partial preservation of LVM [10,36]. Furthermore, Luo et al. (2022) [43] comprehensively reviewed the activation, signaling, and functional roles of *HIF-1* in both physiological homeostasis and pathological conditions. Notably, in breast cancer—one of the malignancies most frequently treated with DOX [44]—overexpression of the *HIF-1α* subunit has been consistently reported. In this context, lower *HIF-1α* levels are associated with more favorable cellular and clinical outcomes, supporting the preservation of left ventricular mass and conferring protection against cardiotoxicity.

As previously discussed, preventive physical training plays a fundamental role in cellular preconditioning for different types of insults, such as those induced by DOX [30,36]. This preparation is manifested both in improved physical endurance—demonstrated by the increase in maximum running speed during the stress test—and in molecular benefits, such as reduced oxidative damage—evidenced by the maintenance of protein carbonylation, lipid peroxidation, SOD and GR levels in the DT group compared to the D group. Thus, these findings reinforce the idea that prior aerobic training acts as cardiac conditioning, preserving redox balance, minimizing mitochondrial dysfunction, and protecting against oxidative cardiac injury induced by DOX [31,36].

Furthermore, SOD acts as an antioxidant defense against oxidative stress by scavenging the superoxide radical [45]. In our study, SOD demonstrates an attempt by cardiac cells to combat oxidative imbalance. Similarly, GR, also elevated in group D, is an enzyme that plays a crucial role in maintaining a reduction environment within cells, protecting against oxidative damage and contributing to the detoxification of peroxides and free radicals [46]. In addition, GR also participates in the regeneration of GSH, which is a radioprotective agent that acts in the maintenance of the non-enzymatic antioxidant pathway [46,47]. Despite the compensatory increase in these antioxidant enzymes in group D, our findings suggest that this mechanism may reflect an overloaded or ineffective cellular system in the face of the oxidative insult promoted by DOX. However, when exposed to physical training (DT group), cardiac cells were able to preserve SOD and GR activity compared to the control groups. Also, in the CT group, the expression of sulfhydryls was high in relation to the other groups. This finding reinforces the relevance of these structures in maintaining protein stability and functionality, since these groups play an essential role in the three-dimensional protein structure and enzymatic activity, in addition to maintaining redox homeostasis [48].

Due to the stimulation of cellular changes, physical exercise initially begins its action on metabolism as a possible insult, as it transiently induces the exacerbated activation of oxidative pathways [49,50]. However, chronic and controlled exposure to exercise promotes an adaptive response, characterized by increased endogenous antioxidant capacity and cellular resistance to subsequent aggressions [49] such as exposure to DOX. Similarly, Morrison et al. (2023) [51] systematically reviewed the effects of resistance exercise on left ventricular structure in healthy adults and concluded that there was a significant increase in chamber size, wall thickness, and LVM after exercise. This study reinforces the benefits of physical exercise observed in cardiac structures undesirably affected by DOX, since DOX-induced cardiotoxicity can be diagnosed by reduced LVEF [36].

In our study, DOX administration resulted in a significant reduction in LVM, reflecting structural cardiac atrophy. It is important to note that other functional cardiac outcomes were not reassessed in the present study, as they were comprehensively evaluated in a previously published investigation from our group using the same experimental model and protocol. In that study, DOX-induced cardiotoxicity led to both a reduction in LVM and deterioration of cardiac contractility, evidenced by decreased LVEF and LVFS [15]. Together, these data reinforce the strong association between myocardial atrophy and functional deterioration, suggesting that the loss of LVM may represent not only a structural consequence of DOX exposure but also a key determinant of cardiac dysfunction.

Importantly, preventive aerobic training attenuated the reduction in LVM, demonstrating a protective adaptation against DOX-induced structural remodeling, even though values did not fully return to physiological levels. This partial preservation of ventricular mass likely reflects greater cardiomyocyte resilience and improved myocardial trophism mediated by exercise-induced preconditioning. From a mechanistic point of view, these benefits may result from the attenuation of oxidative and nitrosative stress, the maintenance of redox balance, and the modulation of *HIF-1*-related stress signaling, which is known to be involved in the regulation of cell survival and death and was attenuated in the DT group. Thus, our results provide further support for the idea that regular aerobic training before chemotherapy acts as an effective cardioprotective intervention, capable of minimizing both structural and functional cardiac impairment associated with doxorubicin-induced cardiotoxicity.

In addition to the previously discussed alterations in cellular integrity and redox balance, our study also reveals significant hematological impacts induced by DOX. We highlight the modulation of erythrocytes, hemoglobin and hematocrit, aspects directly related to oxygen transport and cardiovascular physiology [52]. Antineoplastic therapies, such as DOX, and radiotherapy are well known for their myelotoxic effects, which can suppress bone marrow activity and impair erythropoiesis through the activation of stress- and cell death-related signaling pathways in hematopoietic progenitor cells [53].

Nevertheless, when the animals were exposed to physical exercise alone without DOX treatment, there was stimulation of erythropoiesis, as shown by the increase in the number of erythrocytes in the CT group. Aerobic physical training, especially when regular and moderate, induces an increase in the release of erythropoietin and a greater demand for oxygen, which leads to physiological adaptation [54,55]. The absence of this response in the groups treated with DOX (D and DT groups) suggests that the toxicity of the drug may have suppressed or nullified the erythropoietic stimuli induced by training. Likewise, physical training was not able to normalize—instead only mitigating—the effects of DOX on specific hematological parameters, as shown by the hemoglobin and hematocrit values. That is, DOX compromised not only the number of red blood cells, but also the loading and distribution of oxygen, which exemplifies the common anemic condition in patients after chemotherapy treatment [56].

## 5. Conclusions

Based on our findings, aerobic physical training performed as a preventive strategy prior to DOX exposure exerts a multifaceted cardioprotective effect. Beneficial adaptations were observed across systemic and cardiac-related pathways, including attenuation of oxidative stress, partial modulation of inflammatory signaling, regulation of hypoxia- and stress-related pathways (as reflected by *HIF-1* expression), and preservation of cardiac morpho-functional integrity and LVM. Although exercise did not fully prevent the myelotoxic effects of DOX, it promoted relevant physiological adaptations, particularly under non-chemotherapy conditions, reinforcing its beneficial role in erythropoiesis. Taken together, these results are consistent with the existing literature supporting physical exercise as a complementary intervention during cancer treatment and as an effective preventive strategy against DOX-induced cardiotoxicity. Finally, our findings strengthen the rationale for implementing structured physical training prior to the initiation of chemotherapy as part of multidisciplinary cardio-oncology care protocols.

## 6. Study Limitations

This study did not include tumor-bearing animal models to evaluate the effects of preventive physical training on redox status and LVM, as well as their changes with DOX use, conditions that more closely resemble the clinical context of cancer and chemotherapy. Future studies should also include animal models with a specific age, menopausal status, and concomitant comorbidities (such as obesity, diabetes, and hypertension) that may reproduce the clinical characteristics of most cancer patients. Furthermore, comparative analyses between different exercise modalities (e.g., aerobic vs. resistance training) and intensities are warranted to determine the most effective protocols for mitigating DOX-induced cardiotoxicity and oxidative damage. Regarding inflammation, preventive training partially modulated inflammation, attenuating *IL-1β* mRNA expression without normalizing *IL-6* levels; however, inflammatory mediators were assessed at the mRNA level, which precludes definitive conclusions regarding their protein expression or biological activity. Additionally, the absence of classical apoptotic markers limits conclusions regarding the direct involvement of programmed cell death pathways, and future studies should include specific molecular and histological assessments to better elucidate the role of apoptosis in DOX-induced cardiotoxicity.

## Figures and Tables

**Table 1:** Oligonucleotides used.

Gene	Forward	Reverse
*IL-6*	TCCTACCCCAACTTCCAATGCTC	TTGGATGGTCTTGGTCCTTAGCC
*IL-1β*	CAGAACATAAGCCAACAAGTGGTATT	CACAGGGATTTTGTCGTTGCT
*HIF-1*	TGTTTGATTTTACCCATCCATGTG	TTCTCACTGGGCCATTTCTGT
*iNOS*	GGCTCACGGTCAAGATCCA	ACTCGTACTTGGGATGCTCCAT
*GAPDH*	CTACCCCCAATGTATCCGTTGT	ATGTCATCATACTTGGCAGGTTTC
*CyPA*	GTCAACCCCACCGTGTTCTTC	ACTTGCCACCAGTGCCATTATG

*IL-6* = interleukin-6; *IL-1β* = interleukin-1-beta; *HIF-1* = hypoxia-inducible factor 1; *iNOS* = inducible nitric oxide synthase; *GAPDH* = glyceraldehyde-3-phosphate dehydrogenase; *CyPA* = cyclophilin A.

**Table 2:** General characteristics and blood count of experimental animals.

		Body Mass (g)	ET (km/h)	Erythrocytes (×10^6^/μL)	Hemoglobin (g/dL)	Hematocrit (%)
**C**	**Baseline**	129.3 ± 29.5 ^α^	1.24 ± 0.16 ^α^	N/A	N/A	N/A
	**Final**	268.5 ± 16.2 ^β^	1.17 ± 0.30 ^α^	6.72 ± 1.27 ^α,β^	12.35 ± 2.26 ^α^	37.18 ± 6.80 ^α^
**D**	**Baseline**	137.4 ± 32.8 ^α^	1.07 ± 0.24 ^α^	N/A	N/A	N/A
	**Final**	190.6 ± 31.7 ^δ^	1.08 ± 0.21 ^α^	5.17 ± 1.48 ^δ^	9.68 ± 2.26 ^β^	29.06 ± 6.63 ^β^
**CT**	**Baseline**	121.8 ± 20.2 ^α^	1.14 ± 0.21 ^α^	N/A	N/A	N/A
	**Final**	265.6 ± 17.0 ^β^	2.19 ± 0.40 ^β^	7.40 ± 1.37 ^α^	12.67 ± 2.15 ^α^	38.10 ± 6.41 ^α^
**DT**	**Baseline**	130.6 ±28.1 ^α^	1.17 ± 0.26 ^α^	N/A	N/A	N/A
	**Final**	195.4 ± 30.2 ^δ^	2.03 ± 0.24 ^β^	5.32 ± 1.29 ^β,δ^	10.84 ± 2.16 ^α,β^	32.11 ± 3.02 ^α,β^

Variables are described as mean ± standard deviation; *p* < 0.05 in the ANOVA test was considered significant. Different superscript letters (α, β, δ) indicate statistically significant differences between groups for the same parameter, while identical letters denote no significant difference. ET = maximal exercise tests; C = sedentary control; D = sedentary doxorubicin; CT = trained control; DT = trained doxorubicin; N/A = not assessed.

## Data Availability

The original contributions presented in this study are included in the article/Supplementary Materials. Further inquiries can be directed to the corresponding authors.

## Supplementary Materials

The following supporting information can be downloaded at: https://www.mdpi.com/article/10.3390/cells15050408/s1. Table S1: Individual baseline body weight and left ventricular mass (LVM). *n* = 10 in each group. C = sedentary control; D = sedentary doxorubicin; CT = trained control; DT = trained doxorubicin. The full, unedited echocardiography images are provided in the Supplementary Materials.
