# Supplementary material for: Preventive Aerobic Training Protects Against Doxorubicin-Induced Cardiotoxicity by Preserving Redox Status and Attenuating Cardiac Stress-Related Signaling
Source: Cells. 2026 Feb 26;15(5):408. doi: 10.3390/cells15050408 (PMC12985090; doi:10.3390/cells15050408)

Supplementary Table S1. Individual baseline body weight and left ventricular mass (LVM)

Group	Animal	Body weight (g)	LVM
C	1	142	0.984
C	2	80	0.694
C	3	96	0.898
C	4	103	0.762
C	5	111	0.843
C	6	152	0.839
C	7	124	0.823
C	8	113	0.660
C	9	136	0.782
C	10	171	0.847
CT	1	110	1.734
CT	2	130	0.874
CT	3	139	0.751
CT	4	101	0.868
CT	5	109	0.820
CT	6	124	0.937
CT	7	130	0.846
CT	8	136	0.738
CT	9	107	0.650
CT	10	123	0.808
D	1	137	0.866
D	2	137	0.680
D	3	157	0.888
D	4	93	0.813
D	5	91	0.744
D	6	107	0.645
D	7	112	0.641
D	8	132	0.903
D	9	115	0.947
D	10	138	0.942
DT	1	109	0.899
DT	2	147	0.938
DT	3	100	0.597
DT	4	112	0.844
DT	5	120	0.853
DT	6	140	0.880
DT	7	141	0.822
DT	8	145	0.727
DT	9	129	1.062
DT	10	118	0.719

Table. Abbreviations: C, sedentary control; CT, trained control; D, sedentary doxorubicin; DT, trained doxorubicin; LVM, left ventricular mass.

sedentary control (C)

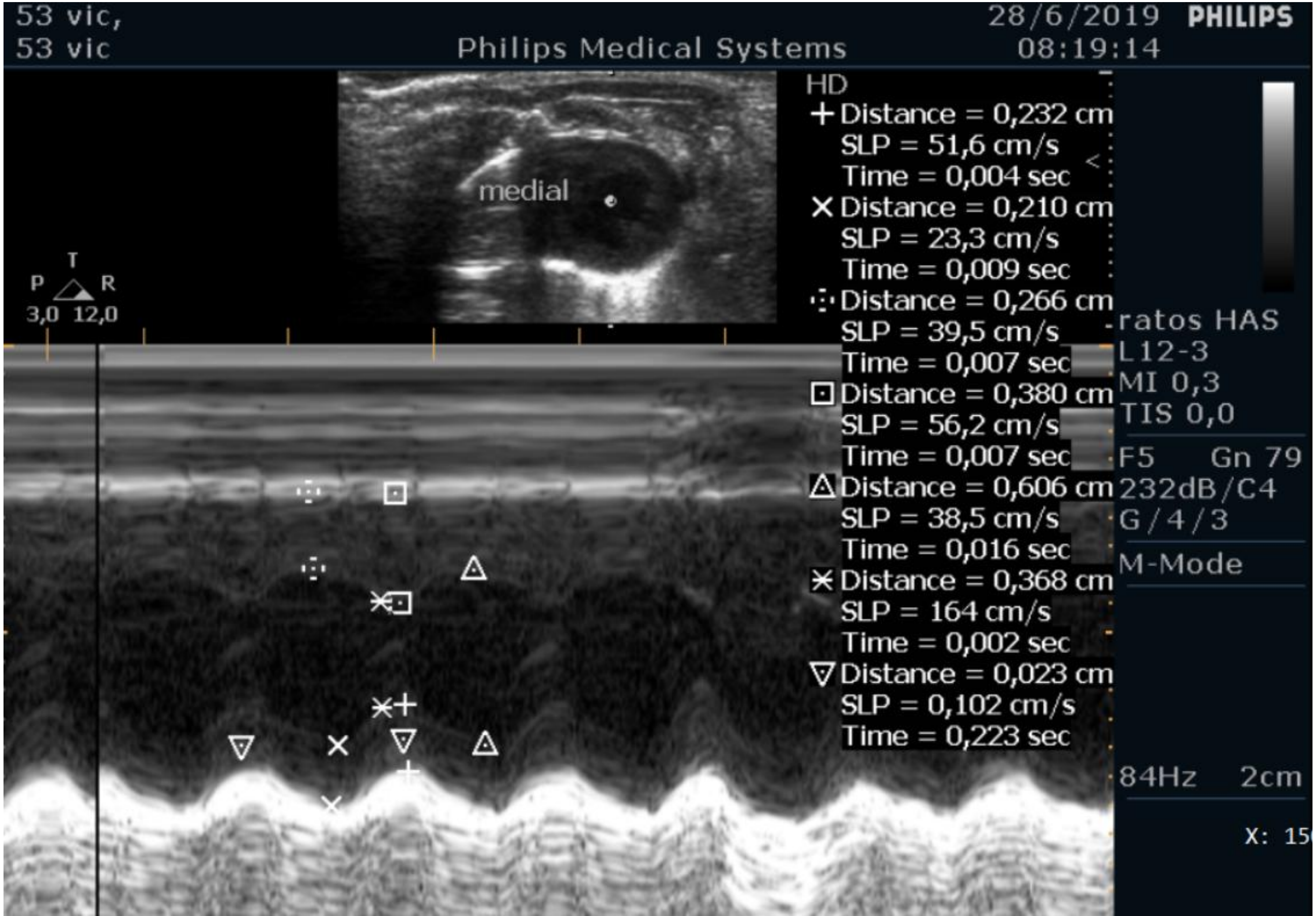

trained control (CT)

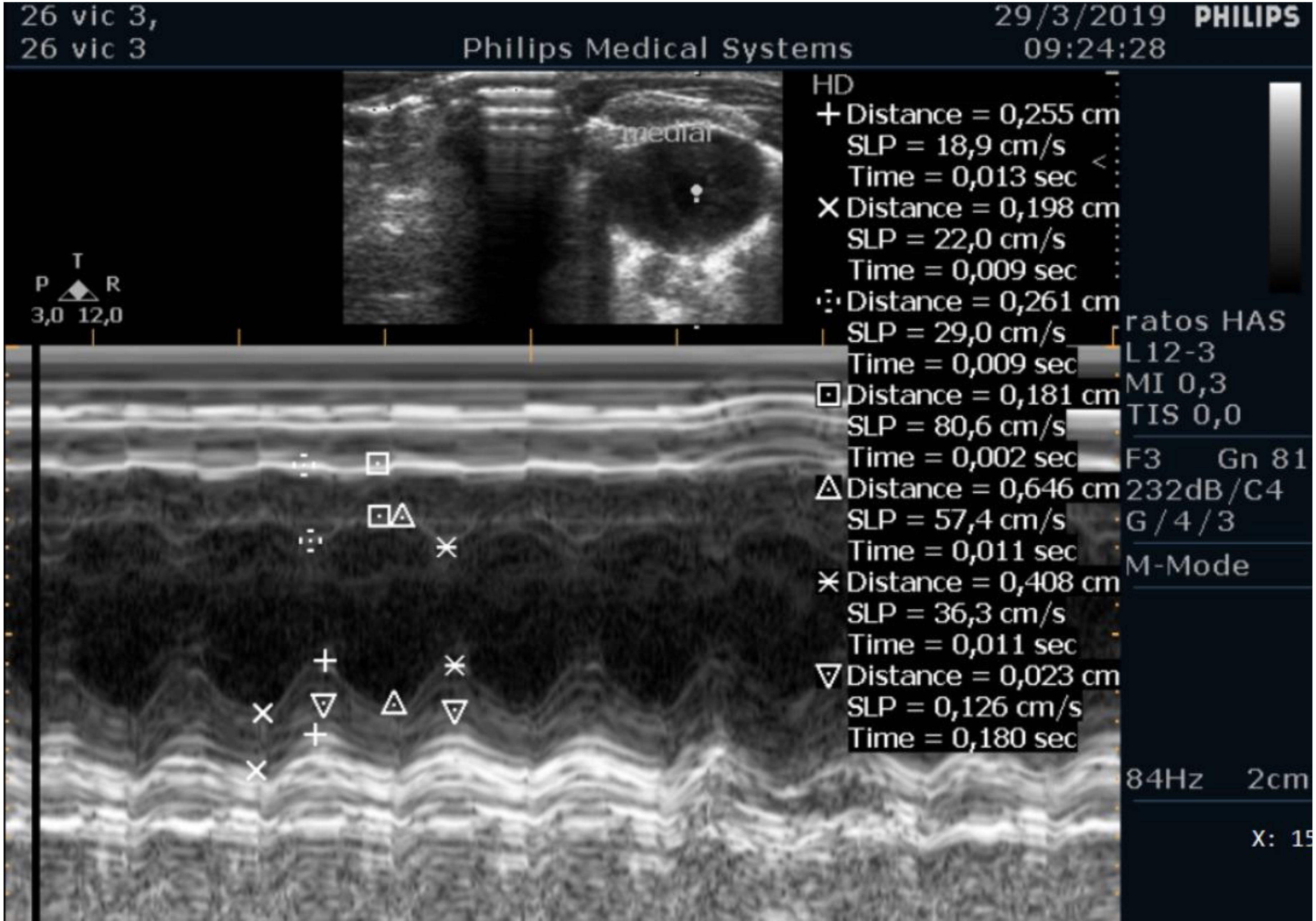

sedentary DOX (D)

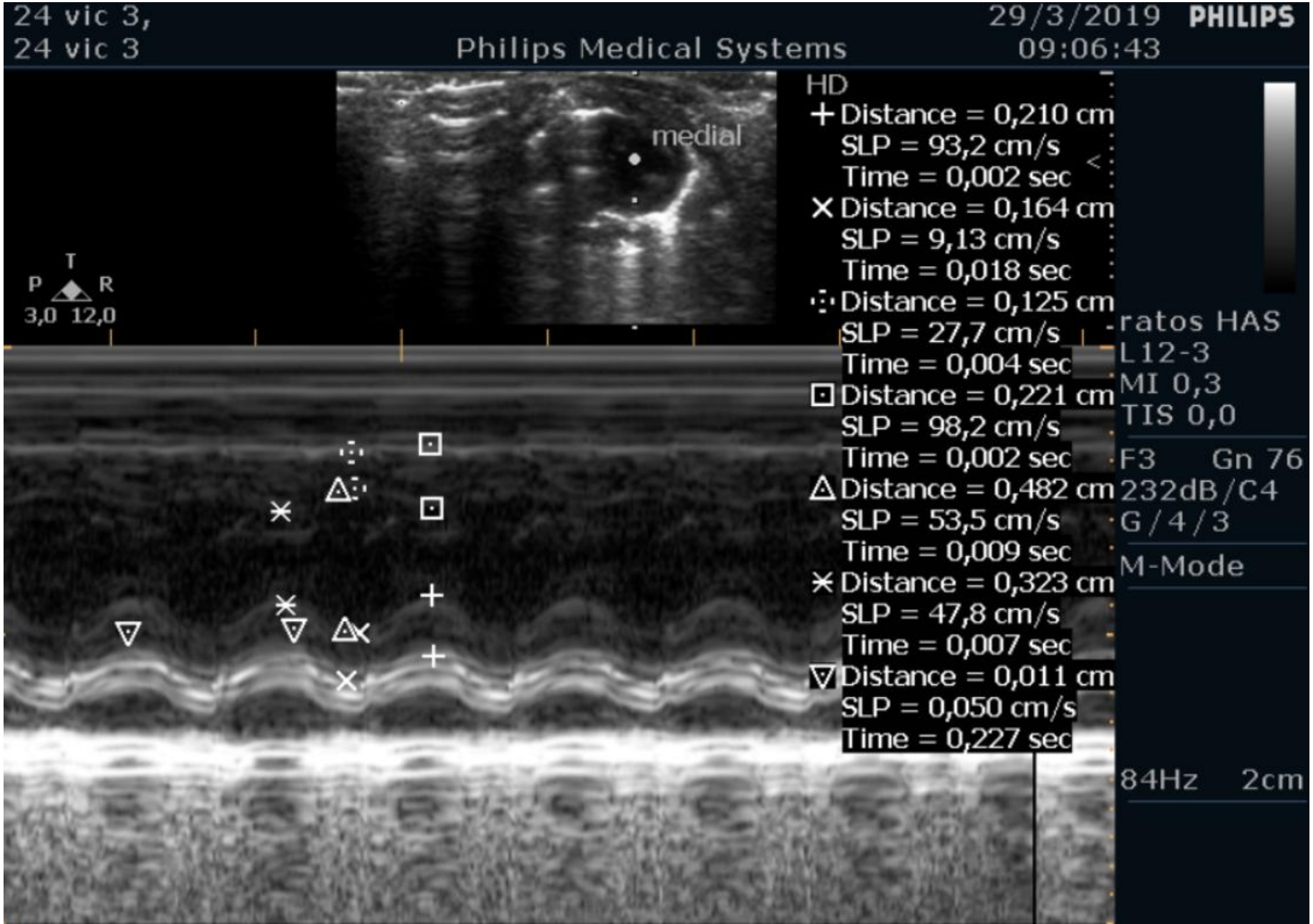

trained DOX (DT)

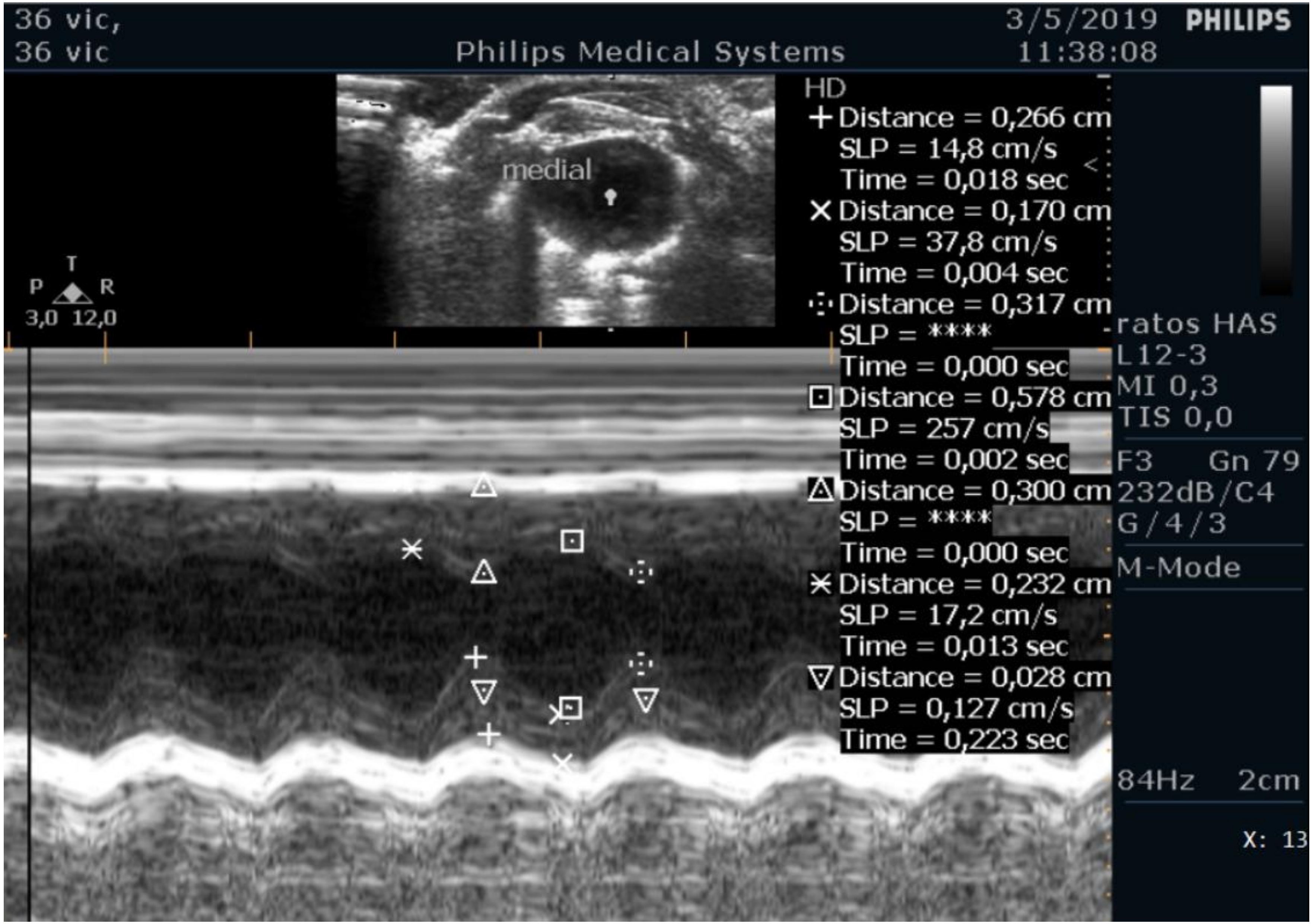

Supplement: Supplementary file 1 [file cells-15-00408-s001.zip › cells-4130373-supplementary.pdf]
